# Investigating the impact of prior COVID‐19 on IgG antibody and interferon γ responses after BBIBP‐CorV vaccination in a disease endemic population: A prospective observational study

**DOI:** 10.1002/hsr2.1521

**Published:** 2023-09-08

**Authors:** Zahra Hasan, Kiran Iqbal Masood, Shama Qaiser, Erum Khan, Areeba Hussain, Zara Ghous, Unab Khan, Maliha Yameen, Imran Hassan, Muhammad Imran Nasir, Muhammad Farrukh Qazi, Haris Ali Memon, Shiza Ali, Sadaf Baloch, Zulfiqar A. Bhutta, Marc Veldhoen, J. Pedro Simas, Syed Faisal Mahmood, Kulsoom Ghias, Rabia Hussain

**Affiliations:** ^1^ Department of Pathology and Laboratory Medicine Aga Khan University Karachi Pakistan; ^2^ Department of Family Medicine Aga Khan University Karachi Pakistan; ^3^ Department of Pediatrics Aga Khan University Karachi Pakistan; ^4^ Center of Excellence in Women and Child Health Aga Khan University Karachi Pakistan; ^5^ Center for Global Child Health Hospital for Sick Children Toronto Canada; ^6^ Instituto de Medicina Molecular, João Lobo Antunes, Faculdade de Medicina Universidade de Lisboa Lisbon Portugal; ^7^ Católica Biomedical Research Center, Católica Medical School Universidade Católica Portuguesa Lisboa Portugal; ^8^ Department of Medicine Aga Khan University Karachi Pakistan; ^9^ Department of Biological and Biomedical Sciences Aga Khan University Karachi Pakistan

**Keywords:** COVID‐19, immunoglobulin G, interferons, T‐lymphocytes, vaccination

## Abstract

**Background and Aims:**

COVID‐19 vaccinations have reduced morbidity and mortality from the disease. Antibodies against severe acute respiratory syndrome coronavirus 2 (SARS‑CoV‑2) have been associated with immune protection. Seroprevalence studies revealed high immunoglobulin G (IgG) antibody levels to SARS‐CoV‐2 in the Pakistani population before vaccinations. We investigated the effect of BBIBP‐CorV vaccination on circulating IgG antibodies and interferon (IFN)‐γ from T cells measured in a cohort of healthy individuals, with respect to age, gender, and history of COVID‐19.

**Methods:**

The study was conducted between April and October 2021. BBIBP‐CorV vaccinated participants were followed up to 24 weeks. Antibodies to SARS‐CoV‐2 Spike protein and its receptor‐binding domain (RBD) were measured. IFNγ secreted by whole blood stimulation of Spike protein and extended genome antigens was determined.

**Results:**

Study participants with a history of prior COVID‐19 displayed a higher magnitude of IgG antibodies to Spike and RBD. IgG seropositivity was greater in those with prior COVID‐19, aged 50 years or younger and in females. At 24 weeks after vaccination, 37.4% of participants showed IFN‐γ responses to SARS‐CoV‐2 antigens. T cell IFN‐γ release was higher in those with prior COVID‐19 and those aged 50 years or less. Highest IFN‐γ release was observed to extended genome antigens in individuals both with and without prior COVID‐19.

**Conclusion:**

We found that IgG seropositivity to both Spike and RBD was affected by prior COVID‐19, age and gender. Importantly, seropositive responses persisted up to 24 weeks after vaccination. Persistence of vaccine induced IgG antibodies may be linked to the high seroprevalence observed earlier in unvaccinated individuals. Increased T cell reactivity to Spike and extended genome antigens reflects cellular activation induced by BBIBP‐CorV. COVID‐19 vaccination may have longer lasting immune responses in populations with a higher seroprevalence. These data inform on vaccination booster policies for high‐risk groups.

## Key points

Baseline seropositivity high in population in those without prior COVID‐19—no antibody waning after vaccination may be due to cross‐reactivity or continued transmission ‐inactivated vaccination induced T cell response to antigens other than Spike providing additional immunogenicity ‐COVID‐19 vaccination may have longer lasting immune responses in populations with a higher seroprevalence—these data inform on vaccination booster policies

## 1 INTRODUCTION

COVID‐19 infections have surpassed 665 million cases, with 6.71 million deaths globally as of February 19, 2023.^1^ Case fatality rates (CFR) from COVID‐19 varied greatly across the globe during the early pandemic waves such as around, March 2020, when observed CFR was 6.2% in Italy, 3.6% in Iran, and 0.79% in South Korea.^2^, ^3^ Vaccinations have had a major impact on controlling both morbidity and mortality from COVID‐19.^4^

The Spike glycoprotein of severe acute respiratory syndrome coronavirus 2 (SARS‐CoV‐2) is highly immunogenic and antibodies to Spike are associated with protective immunity against the virus.^5^, ^6^ Humoral responses against SARS‐CoV‐2 are driven both by natural infection and COVID‐19 vaccinations.^5^, ^6^, ^7^ Recognition of Spike and nucleocapsid proteins by T cells is associated with clearance of SARS‐CoV‐2.^8^

COVID‐19 vaccines include formulations based on messesnger RNA (mRNA) expression of Spike, adenovirus vector‐based vaccines, and inactivated vaccine types.^9^ BBIBP‐CorV prepared by Beijing Bio‐Institute of Biological Products Co. Ltd. (Sinopharm) is an aluminum‐hydroxide‐adjuvanted, inactivated whole‐virus vaccine.^10^ By February 19, 2023, 575.8 million doses of BBIBP‐CorV were delivered worldwide.^11^ However, there is limited data on inactivated vaccines such as those administered in many low‐middle income countries.

Pakistan has a population of greater than 200 million, and 1.58 million COVID‐19 cases, with 30,641 related deaths reported (February 19, 2023).^1^ However, COVID‐19 related morbidity was relatively low during the pandemic and the CFR did not rise above 2% even before the introduction of vaccinations.^12^ COVID‐19 vaccinations in Pakistan were rolled out in February 2021, whereby BBIBP‐CorV was one of the primary vaccines administered.^13^ By February 19, 2023, 162.2 million people had received vaccinations, with 73.4% of the population administered at least their first dose of a two‐dose vaccine regimen.^1^

Studies from the early pandemic period of 2020 in Pakistan showed antibody seroprevalence to range between 15% and 21% in some areas of Karachi, the largest urban center in Pakistan.^14^ By December 2020, we observed immunoglobulin G (IgG) antibodies to Spike to be present in greater than 50% of unvaccinated healthy blood donors.^15^ During 2021, Pakistan experienced three pandemic waves which were dominated first by Alpha, then Delta and subsequently Omicron variants.^16^ Given the role of antibodies in protection against SARS‐CoV‐2, and that IgG antibodies are induced both by viral infection and vaccinations, it is important to understand seropositivity in both these context. Here we investigated IgG antibody responses to Spike and receptor‐binding domain (RBD) and also, interferon (IFN)‐γ responses to whole blood stimulation by SARS‐CoV‐2 antigens in cohort of healthcare associated individuals vaccinated with BBIBP‐CorV. We also determined the effect of prior COVID‐19 infection on immune responses after BBIBP‐CorV vaccination and those of age and gender on host immunity.

## 2 MATERIALS AND METHODS

### 2.1 Study description

This was a prospective observational study with a consecutive convenience sampling design. Information regarding the study was circulated at the institution and interested volunteers were encouraged to contact the study team, who informed them about the study and recruited individuals with their consent. Study participants included healthcare workers, other Aga Khan University (AKU) employees and their family members who volunteered for the study. Subjects were recruited between April and October 2021. Inclusion criteria were males and females aged over 18 years. Exclusion criteria; individuals with history of chronic infections such as, hepatitis viruses, tuberculosis, or autoimmune conditions.

BBIBP‐CorV vaccination was administered as per guidelines of the National Covid Operation and Command (NCOC), Government of Pakistan.^17^ The vaccine route was an intramuscular injection in the deltoid area. The time interval between the first and second doses of BBIBP‐CorV was 4 weeks as per manufacturer's recommendations.^10^

We recruited 312 adult study subjects who had already received their first dose of BBIPP‐CorV vaccination at the AKU Hospital COVID‐19 Vaccination Center. They were subsequently followed up at the study time points of 4, 8, 12, 20 and 24 weeks after first dose of vaccination.

A verbal history of prior COVID‐19 infection was taken at the time of recruitment. Positive antibody results if available were also documented. There was no bias in selection of cases based on any prior COVID‐19 history.

### 2.2 Sample collection

Serum samples were collected for measurement of IgG antibodies at 4, 8, 12, 16, 20 and 24 weeks postvaccination. The number of samples given by each participant varied. In all, 312 participants submitted ≥1 test; 248 underwent ≥2 tests, 151 gave ≥3 samples and 41 gave 4 samples (Supporting Information: Figure 1). Whole blood for interferon gamma release assay (IGRA) was collected for a subset of 99 participants between 20 and 24 weeks after vaccination. The range between 20 and 24 weeks was used as this was the period associated with waning of immunity after vaccinations.^18^

### 2.3 Recombinant proteins

Recombinant Spike and RBD proteins were produced by the laboratory of Prof. Paul Alves, iBET, NOVA ITQB University, Portugal. The proteins were extensively characterized and found to be both stable and consistent for use in serological assays.^19^, ^20^

### 2.4 ELISA for IgG to spike and RBD

The assay used was based on the protocol developed by the laboratory of Prof. Florian Krammer^21^, ^22^ and received FDA authorization.^23^ The enzyme linked immunosorbent assay (ELISA) has been described previously.^6^, ^15^, ^22^ The cut‐off was established by calculating the mean +2 SD (optical density [OD]: 0.5 at 450 nm) for IgG measurements of Spike and RBD in a prepandemic controls data set. Briefly, SARS‐CoV‐2 Spike and/or RBD protein were used to coat plates with 50 µL of Spike or RBD protein at a concentration of 2 µg/mL in PBS. Wells were blocked, incubated with serum, stained with secondary antibody conjugated with Horse Radish Peroxidase (HRP) and read at as OD units at 450 nm.

### 2.5 QuantiFERON SARS‐CoV‐2 assay

Five mililitre of whole blood was collected in a lithium heparin collection tube. QuantiFERON ELISA (Cat. No. 626410) and QuantiFERON (QFN) SARS‐CoV2 RUO Starter + Extended Pack (Cat. No. 626915); Qiagen was used as the IGRA. This assay consists of three Antigen tubes, SARS‐CoV‐2 Ag1, Ag2 and Ag3, that uses a combination of proprietary antigen peptides specific to SARS‐CoV‐2 to stimulate lymphocytes involved in cell‐mediated immunity in heparinized whole blood. The QFN SARS‐CoV‐2 Ag1 tube contains CD4 + epitopes derived from the S1 subunit (RBD) of the Spike protein, the Ag2 tube contains CD4 + CD8 epitopes from the S1 and S2 subunits of the Spike protein and the Ag3 tube consists of CD4 + CD8 + epitopes from S1 and S2, and epitopes from M and the rest of the genome. Plasma from stimulated samples were used for detection of IFN‐γ using an ELISA to determine quantitative results (IFN‐γ concentration in IU/mL). The cut‐off (elevated response) was defined as a value at least 0.15 IU/mL greater than the background IU/mL value from the QFN‐SARS‐CoV‐2 Nil tube.

### 2.6 Statistical analysis

Scatter plots depicting IgG levels were drawn using GraphPad PRISM 5.0 software. A nonparametric analysis of significance between IgG levels between groups was determined using Mann–Whitney *U* test (MWU). A comparison of IgG values across different time periods was determined using the Kruskal–Wallis (KW) test (two‐tailed). Correlation between the Spike and RBD IgG levels was determined using the Spearman's rank correlation test. A *p* ≤ 0.05 was considered as significant.

The Statistical Package for the Social Sciences version 24.0 (SPSS Inc., 2013) was used to carry out descriptive statistics of participants for demographic variables (gender, age and prior COVID‐19). *χ* ^2^ test was used to compare the frequencies of IgG antibodies with respect to age groups (either less than or equal to 50 years or those above 50 years), gender and H/O COVID infection as variables. The threshold of significance was a *p* ≤ 0.05.

A multivariate analysis was conducted with age, gender, and H/O COVID adjusted to the frequency of positive IgG responses determined in each condition. An odds ratio (OR) was used to determine significance in the multivariate analysis, using Ref interval of male gender, and no H/O COVID in respective analysis. 95% CI was determined for the determining significance at a *p* ≤ 0.05.

## 3 RESULTS

### 3.1 Description of study

BBIBP‐CorV (Sinopharm) vaccinations were the first to be administered in Pakistan during February 2021 including, at the Aga Khan University Hospital which was designated a COVID‐19 Vaccination Center by the Department of Health, Government of Sindh. This study was initiated in March and blood samples were collected between April and October 2021. We included participants who had received their first dose of BBIBP‐CorV vaccine between February and June 2021 and followed them for 24 weeks. This was when Pakistan was facing pandemic surges associated with SARS‐CoV‐2 Alpha and Delta variants.^16^ It was a time when there were frequent restrictions to travel both within and outside the country, supported by lockdowns. Not all study participants were able to submit study samples at the requested frequency.

The age range of study subjects was 20–101 years with a mean age of 40.7 ± 16.5 years (Table 1). Seventy four percent of subjects (*n* = 231) were aged <50 years, 26% (*n* = 81) ≥50 years; while 63% were females. Eighty‐nine individuals (28.5%) had a history of COVID‐19 (H/O COVID) whilst 223 individuals did not (no H/O COVID).

**Table 1:** Characteristics of study subjects.

Group	*n* (%)	Females	Males
Total	312 (100)	196 (62.8%)	116 (37.2%)
≤50 years (*n*, %)	231 (74)	154 (66.7)	77 (33.3)
>50 years (*n*, %)	81 (26)	42 (52)	39 (48)
History of COVID			
Yes (*n*, %)	89 (28.5%)	60 (67.4)	29 (32.6)
Age (years), mean (SD)			
Total	**44 (14)**		
COVID prevaccination	34.4 (10.9)		
COVID postvaccination	44.2 (13.8)		
Prevaccination COVID‐19 (*n*, %)	**68** (**21.8%)**	**47** (**69.1)**	**21** (**30.9)**
Age‐wise			
≤50 years	59 (86.8)	40 (85.1)	19 (90.5)
>50 years	9 (13.2)	7 (14.9)	2 (9.5)
Postvaccination COVID‐19 (*n*, %)	21 (6.7)	13 (62)	8 (38)
Age‐wise			
≤50 years	13 (62)	4 (30.8)	9 (69.2)
>50 years	8 (38)	4 (50)	4 (50)

Of those with H/O COVID; 68 (21.8%) had COVID‐19 before enrollment in the study, 21 (6.7%) individuals suffered COVID‐19 postvaccination and during the study period. None developed COVID‐19 during the follow‐up period of the study. Of those with H/O COVID prevaccination; 59 (86.8%) individuals were ≤50 years and 9 (13.2%) were >50 years. Thirteen (62%) participants were ≤50 years, with 8 (38%) >50 years. Individuals with H/O COVID prevaccination were younger (*p* = 0.0039) as compared with those who suffered COVID postvaccination.

When the number of each gender was studied within each subgroup (H/O COVID), it was an equivalent number of males and females present in those aged 50 years and below, and those aged above 50 years.

For those who had H/O COVID prevaccination, the median period was 23 weeks before enrollment (range 3–56 weeks). For with postvaccination H/O COVID postvaccination, the median period of developing infection was 16 weeks (range: 12–25 weeks), after the first dose of BBIBP‐CorV.

### 3.2 Effect of history of COVID‐19 on IgG antibody after BBIBP‐CorV vaccination

**Figure 1:**
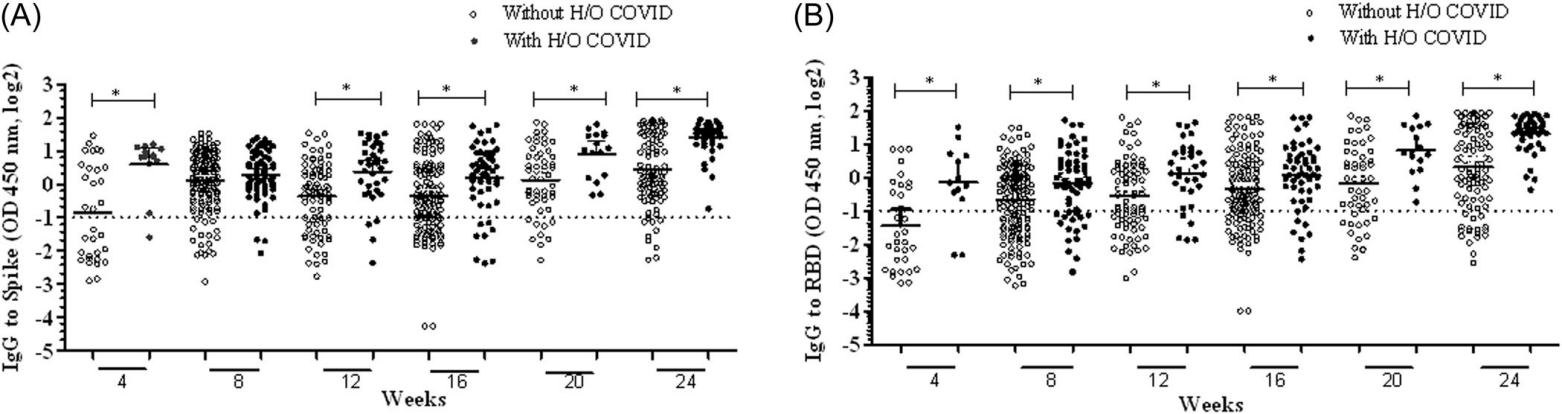
Effect of prior COVID‐19 on IgG responses to Spike and RBD after BBIBP‐CorV vaccination. IgG antibodies were determined in sera of individuals who either had a history of COVID (*n* = 89, shown as closed circles) or did not (*n* = 223, shown as open circles) before vaccination. IgG levels at 4, 8, 12, 16, 20, and 24 weeks postvaccination are depicted to (A) Spike and (B) RBD. The cut‐off for positive responses at 0.5 OD 450 nm is indicated by a dotted horizontal line. Graphs show the geometric mean as a horizontal bar with 95% confidence interval indicated by upper error bars. *p ≤ 0.05. IgG, immunoglobulin G; RBD, receptor‐binding domain.

To determine the effect of prior infection, we compared responses in participants with and without H/O COVID, studying IgG levels increased at 4, 8, 12, 16, 20 and 24 weeks after vaccination. BBIBP‐CorV vaccination resulted in a subsequent increase in IgG antibodies to Spike between 4 and 24 weeks in individuals with H/O COVID (*p* < 0.001) and also in those without H/O COVID (*p* < 0.001, Figure 1A, KW test).

IgG antibody levels to RBD also increased significantly across the study period of 24 weeks, both with and without H/O COVID‐19 showing statistical significance of *p* < 0.001, respectively (Figure 1B). In participants with H/O COVID, we found the magnitude of IgG levels to Spike to be higher at 4 weeks (*p* < 0.001), 12 weeks (*p* < 0.001), 16 weeks (*p* < 0.001), 20 weeks (*p* < 0.001), and 24 weeks (*p* < 0.001) after vaccination, as determined using MWU analysis. The magnitude of IgG levels to RBD at 4 weeks (*p* < 0.001), 8 weeks (*p* = 0.004), 12 weeks (*p* < 0.001), 16 weeks (*p* < 0.006), 20 weeks (*p* = 0.002), and 24 weeks (*p* < 0.001) as per MWU analysis, was also was higher in participants with H/O COVID as compared with those who did not have H/O COVID. We did not observe any waning of IgG antibody responses over the study period.

**Figure 2:**
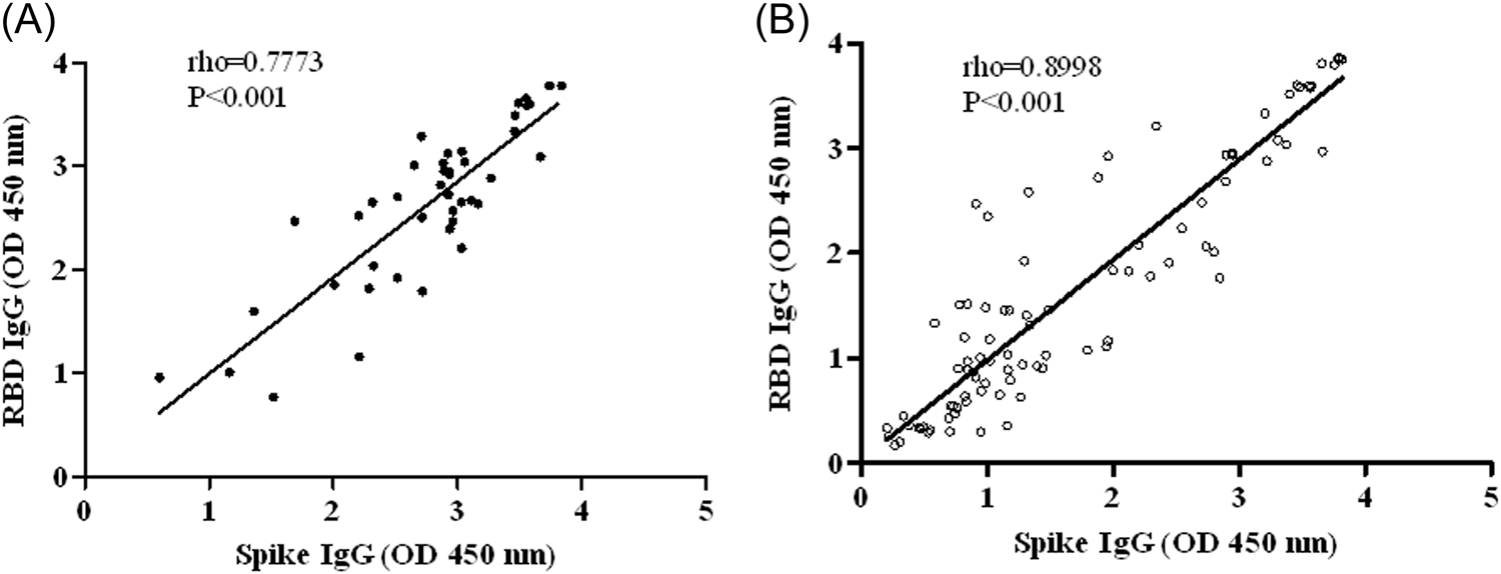
Concordance between IgG responses to Spike and RBD after vaccination. IgG antibody levels to Spike and RBD measured after 24 weeks of vaccination were correlated with each other in subjects (A) with a history of COVID and (B) without a history of COVID. Spearman's rank correlation analysis was performed and shows *r*, spearman's *⍴*, with >0.5 indicating a significant positive correlation between the parameters tested. *p* ≤ 0.05 indicates statistically significant association. IgG, immunoglobulin G; RBD, receptor‐binding domain.

We next determined the relationship between IgG to Spike and RBD. For this we analyzed antibody data of participants collected 24 weeks after BBIBP‐CorV vaccination. A significant correlation was found between IgG levels to Spike and RBD in those with H/O COVID (*p* < 0.001, *⍴* = 0.7773, Spearman's rank correlation), Figure 2A. Similarly, a concordance between IgG to Spike and RBD was found in those with no H/O COVID (*p* < 0.001, *⍴* = 0.8998), Figure 2B. Although a positive correlation between IgG to Spike and RBD was found in individuals both with and without H/O COVID, the spread of IgG levels (denoted by OD values) differed between the groups. Those with H/O COVID displayed a higher trend of IgG levels than those without H/O COVID. The latter had a wider range of IgG levels, with more individuals with IgG values OD < 1.0.

### 3.3 Effect of H/O of COVID, age and gender on antibody responses

We determined the seropositivity of participants and then investigated the effect of confounders that affect vaccination induced immunity, studying the dynamic effect of age and gender using a multi‐variate model adjusted for age (≤50, >50 years), gender and H/O COVID‐19 on antibody responses to Spike and RBD.

**Figure 3:**
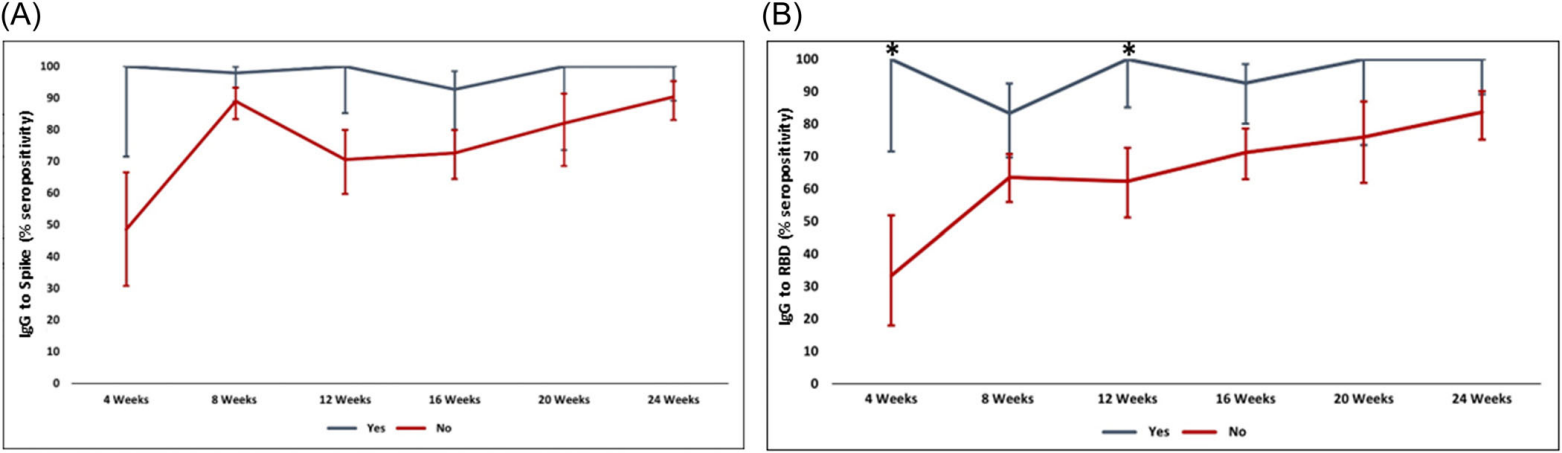
Effect of history of COVID‐19 on IgG seropositivity to Spike and RBD after BBIBP‐CorV vaccination. IgG antibodies were determined in sera of individuals who either had H/O of COVID (indicated as yes, gray line) or did not (indicated as no, red line). IgG seropositivity was determined at 4, 8, 12, 16, 20, and 24 weeks postvaccination. Graphs show seropositivity to (A) Spike and (B) RBD. IgG seropositivity is shown with 95% confidence intervals with error bars at ±2SD. *p ≤ 0.05 between groups. IgG, immunoglobulin G; RBD, receptor‐binding domain.

After adjusting for age and gender, Spike IgG seropositivity between 4 and 24 weeks after BBIBP‐CorV vaccination was compared in those with and without H/O COVID, determining the OR of increasing seropositivity depending on the variable, with 95% CI. Seropositivity to Spike was raised at 4 weeks in those with H/O COVID (*p* = 0.021, Figure 3A, Supporting Information: Table 2). No difference was observed in seropositivity determined at later time points. Similarly IgG seropositivity to RBD was higher at 4 (*p* = 0.007) and 12 (*p* = 0.034) weeks after BBIBP‐CorV vaccination in those with H/O COVID (Figure 3B, Supporting Information: Table 3). No difference was observed at later time intervals 20 and 24 weeks after vaccination.

**Figure 4:**
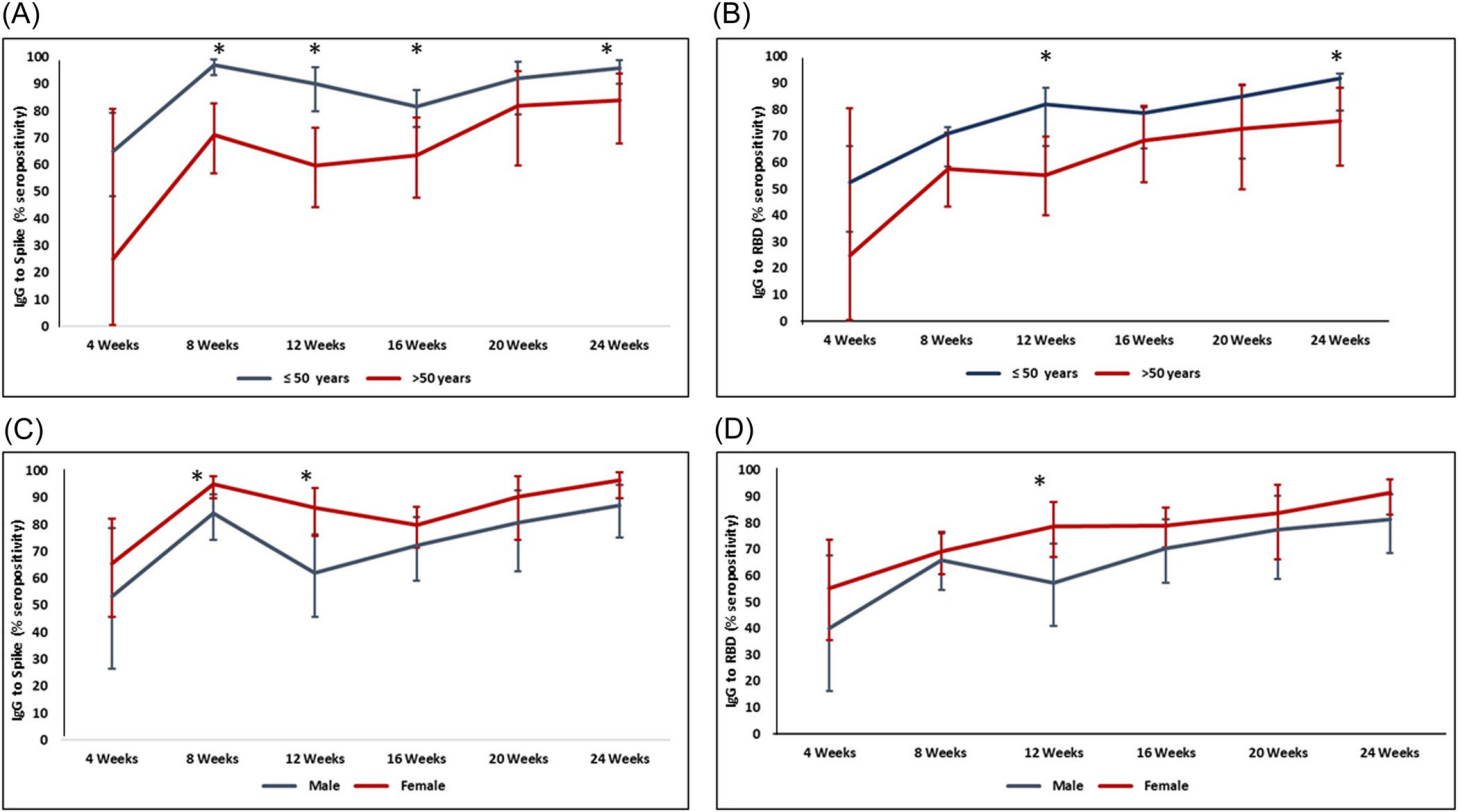
Effect of age and gender on IgG responses to Spike and RBD after BBIBP‐CorV vaccination. Graphs compare IgG seropositivity between individuals in individuals tested at 4, 8, 12, 16, 20, and 24 weeks postvaccination. Comparisons were made based on aged 50 years and below (gray line) to those aged above 50 years (red line) for IgG to Spike (A) and RBD (B). Comparison based on gender (male, gray line and female, red line) for IgG to Spike (C) Spike and RBD (D). *p ≤ 0.05 at each particular time point between groups. The data is indicated as 95% confidence intervals with error bars at ±2SD.

We next investigated the effect of age by analyzing the frequency of seropositive individuals at each time interval stratified by age. IgG seropositivity to Spike greater in those ≤50 years as compared with older individuals at; 8 weeks (*p* < 0.001), 12 weeks (*p* < 0.001), and 16 weeks (*p* = 0.015) weeks postvaccination (Figure 4A, Supporting Information: Table 2). IgG seropositivity to RBD was higher in those aged ≤50 years at 8 and 24 weeks postvaccination (*p* = 0.003 and *p* = 0.015, respectively; Figure 4B, Supporting Information: Table 3).

We observed an effect of gender on the frequency of IgG seropositivity to Spike; with higher responses among females at; 8 weeks (*p* = 0.01) and 12 weeks (*p* = 0.005) weeks postvaccination (Figure 4C). IgG seropositivity to RBD was higher in females than males at 12 weeks (*p* = 0.02) postvaccination (Figure 4D).

### 3.4 SARS‐CoV‐2 antigen induced T cell responses to epitopes of CD4 and CD8

We aimed to understand the longevity of T cell IFN‐γ response induced BBIBP‐CorV, studying responses to SARS‐CoV‐2 antigens. Previous studies had shown T cell activation induced by BBIBP‐CorV vaccination to be present in individuals 12 weeks after their second dose^24^ however, it was not known how long the T cell response persisted. We chose the period of 24 weeks after vaccination was when other studies had observed waning of immunity after COVID‐19 vaccination.^18^ Particularly, this was important in the context of recommendations for booster vaccinations. Further, we had observed no statistical difference between IgG antibody levels of individuals tested after BBIBP‐CorV vaccination when these time points were compared (Figure 1).

We used the QFN SARS‐CoV‐2 assay to test participants between 20‐ and 24‐weeks postvaccination. We compared T cell IFN‐γ release in 99 participants, of whom 15 had H/O COVID and 84 had no H/O COVID. Participants had a mean of 45 years SD 16 years (range: 19–82 years). Sixty‐eight (68.7%) percent were aged below or equal to 50 years while 31 (31.3%) were aged 50 years and above. Those with H/O COVID comprised 67% females, Table 2. Those without H/O COVID comprised 58.6% females.

**Table 2:** Individuals with prior COVID‐19 show increased IFN‐γ responses to SARS‐CoV‐2 antigens.

Parameters	Total (*n*)	COVID‐IGRA positive (*n*)	COVID‐IGRA negative (*n*)	% IGRA positive
	99	*37*	*62*	37.4
Age (years)				
≤ 50	68	30	38	44.1
>50	31	7	24	22.5
Sex				
Male	41	16	25	39
Female	58	21	37	36.2
History of COVID				
H/O COVID	15	10	5	66.6
No H/O COVID	84	27	57	32.1

*Note*: IFN‐γ responses in whole blood to SARS‐CoV‐2 antigens as per the QuantiFERON SARS‐CoV‐2 assay (Qiagen) are depicted. H/O, history of COVID‐19.

Measurement of T cell activation in the overall group showed the release of IFN‐γ in stimulated supernatants in response Ag1 (*p* = 0.003), Ag2 (*p* < 0.001), and Ag3 (*p* < 0.001), Supporting Information: Figure 2A and Table 1. Overall, 37 participants (37.4%) had a positive IFN‐γ response to one or more antigens (Table 2). According to different antigens, positive IFN‐γ responses were observed in 16% of individuals to Ag1, 24% of individuals to Ag2 and 33% of individuals to Ag3, Supporting Information: Figure 2B.

**Figure 5:**
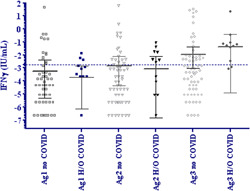
Effect of prior COVID‐19 on the T cell IFN‐γ response after BBIBP‐CorV vaccination. IFN‐γ was measured in stimulated whole blood supernatants of donors 20–24 weeks postvaccination using the QFN SARS CoV‐2 SARS‐CoV‐2 assay, containing Ag1, Ag2, and Ag3 tubes. Tests were conducted in participants with no H/O COVID (*n* = 84) or with H/O COVID (*n* = 15). Graphs show IFN‐γ measured in stimulated supernatants for each group. Data is depicted as the geometric mean, horizontal line with 95% confidence interval indicated by error bars. IFN‐γ, interferon γ.

Next, we compared antigen stimulated T cell IFN‐γ responses in those who did not (*N* = 84) and those with H/O COVID (*N* = 15). As seen in Figure 5, no difference was observed between IFN‐γ levels stimulated by any of the three antigens between the two groups.

We investigated IFN‐γ responsiveness as per age and gender of the donors (Table 2). IFN‐γ responses to SARS‐CoV‐2 antigens were compared between those ≤50 years, with those >50 years. Reactivity to SARS‐CoV‐2 antigens was 44% in the younger age group as compared with 22.5% in the older age group. No difference was seen between T cell responses to Ag1, Ag2, or Ag3 between males and females.

**Figure 6:**
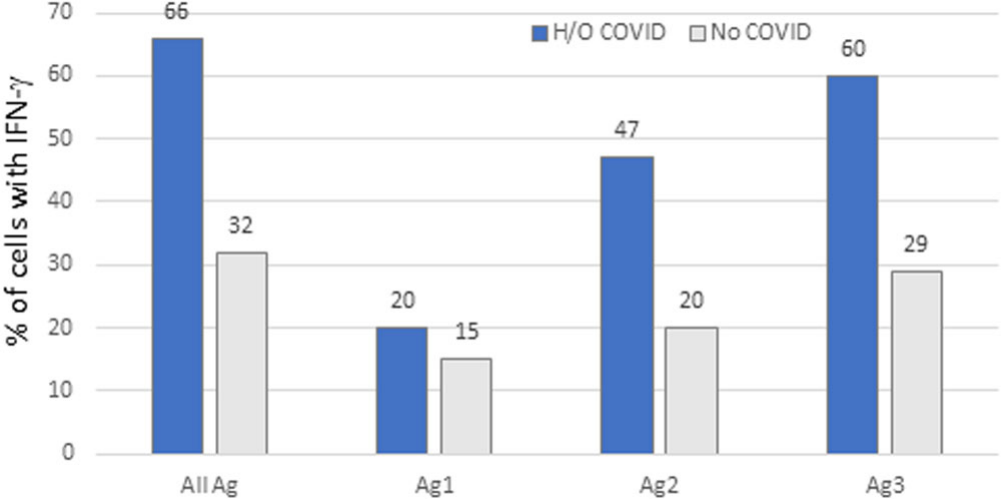
Increased frequency of IFN‐γ from T cells in those with H/O COVID‐19 and BBIBP‐CorV vaccination. IFN‐γ was measured in stimulated whole blood supernatants of donors 20–24 weeks postvaccination using the QFN SARS CoV‐2 SARS‐CoV‐2 assay, containing Ag1, Ag2, and Ag3 tubes. Tests were conducted in participants with no H/O COVID (*n* = 84) or with H/O COVID (*n* = 15). Graphs show IFN‐γ measured in percentage of individuals with reactive T cells to each Ag stimulation. IFN‐γ, interferon γ.

Overall, we observed that after 24 weeks of vaccination, 32.5% of participants with no H/O COVID showed IFN‐γ reactivity to T cell antigens, as compared with reactivity in 66% of participants with H/O COVID (Figure 6). Cellular reactivity induced by Ag1 was found to be comparable in participants with and without H/O COVID. However, reactivity to both Ag2 (47% in H/O COVID, 20% in no COVID) and Ag3 (60% in H/O COVID, 29% in no COVID) was greater in those with H/O COVID.

## 4 DISCUSSION

Our study shows that BIBBP‐CorV vaccination induced greater IgG antibody to Spike and RBD in those with H/O of COVID, aged younger than 50 years or were female. We found T cell IFN‐γ responses to Spike and extended genome antigens was greater in those with prior COVID‐19 or aged younger than 50 years. The work provides insights into antibody and T cell reactivity after administration of an inactivated virus vaccine in a highly disease endemic population.

We observed a significant increase in IgG antibodies to both Spike and RBD up until 24 weeks postvaccination without waning. This is in contrast to a study from Sri Lanka that showed a waning of BBIBP‐CorV induced antibodies by 12 weeks after vaccination.^25^ A case–control study of vaccine efficacy against SARS‐CoV‐2 variants using BNT162b2 in Qatar also showed protective antibody levels to decrease after vaccination.^26^ The absence of antibody waning here may be a consequence of continued SARS‐CoV‐2 exposure in the community, boosting cross‐reactive responses. By February 2021, a study in healthy blood donors conducted in Karachi showed seropositivity to be, 57% to Spike and 32% to RBD.^15^ The period covered by this study (April–October 2021) coincided with the waves caused by Alpha and then Delta variants, leading to high transmission rates.^16^

Full vaccination period is considered at 2 weeks or more after the second dose of a two‐dose vaccine regimen. A study from Sri Lanka by Jeewandara et al. showed that BBIBP‐CorV vaccination resulted in 95% seroconversion at 6 weeks and also found antibodies associated with neutralizing activity to SARS‐CoV‐2.^27^ Earlier, we reported IgG to RBD measured in both COVID‐19 convalescent and healthy individuals to be associated with neutralizing activity to SARS‐CoV‐2.^28^ Here, we found IgG seropositivity at 8 weeks postvaccination in participants without a history of COVID‐19 to be lower at, 89.1% to Spike and 64.9% to RBD. Our data correlates with previous reports from Pakistan that showed 78% of individuals to display antibody responses to RBD after vaccination with BBIBP‐CorV.^29^ Another consideration that may impact the quality of neutralizing antibodies generated by BBIBP‐CorV could be the effect of inactivation of Spike protein in the formulation. Cai et al. have shown that the antigenic epitopes presented by the glycoprotein are dependent on its structural integrity.^30^ Therefore, virus inactivated vaccines may contain proteins that do not have the RBD domain in the open conformation required to serve as effective antigenic epitopes.

BNT162b2 mRNA and ChAdOx vaccinations have been shown to effectively induce IgG antibodies to Spike and neutralizing antibodies, within 14 days of the second dose, or full vaccination.^18^, ^31^ The slower rise in BBIBP‐CorV‐induced IgG to RBD we observed correlates with earlier reports.^32^

BBIBP‐CorV vaccination‐induced IgG seropositivity to Spike and RBD was higher in those aged 50 years and below as compared with those above 50 years. These data correlate with those reported by Ferenci et al. from Hungary who showed that RBD‐specific antibody responses after two doses of BBIBP‐CorV were present in 90% of cases below 50 years but were reduced in those who were older.^33^ BBIBP‐CorV vaccination data from Sri Lanka also shows reduced immune responses in individuals aged 60 years and above.^25^

A possible explanation for the difference observed between early postvaccination responses between younger and older age groups may be related to T independent B cell expansion. T cell activation may occur earlier in the younger age group (<50 years). T follicular helper cell independent expansion has been shown to occur in response to SARS‐CoV‐2 infection in mice, resulting in high affinity antibodies.^34^ In older age groups, T cell activation is compromised^35^ and therefore, the antibody response may drop with removal of antigen antibody complexes. However, the T independent responses continue to produce IgG antibodies as likely indicated by slow rise of IgG antibodies to RBD.^36^

We found that seropositivity of IgG to spike and RBD in females was greater than males after vaccination. A comparison of antibody responses to ChAdOx1 has been shown to induce higher levels in females than males.^31^ Sex‐specific differences related to COVID‐19 have been observed between males and females, with increased COVID‐19 morbidity in males.^37^, ^38^

T cell immunity is associated with long term memory responses and is the hallmark of protection induced by natural infections and vaccines. The T cell responses show durable and polyfunctional virus specific memory CD4 and CD8+ T cells in infected patients up to 8 months after infection, and specificity was observed to a range of SARS‐CoV‐2 antigen.^39^ We used a whole blood based assay to measure T cell activation, this IFN‐γ release assay has been used to measures memory responses in COVID‐19 convalescent individuals^40^ and also determine T cell responses to BNT2162b2 mRNA vaccination responses.^41^

We found that approximately one‐third of individuals had IFN‐γ reactivity to SARS‐CoV‐2 antigens 24 weeks after vaccination, and which was significantly increased those with a prior history of COVID‐19. We observed the strongest T cell responsiveness to antigens included Spike, Membrane and Envelope proteins. This highlights the recognition of conserved or cross‐reactive epitopes by T cells to inactivated vaccines such as BBIBP‐CorV. Enhanced cellular immunity in those with COVID infection history and vaccination has previously been shown to be the case for mRNA vaccines.^42^, ^43^

Overall, we found both antibody and T cell responses to behigher in those who had a history of COVID‐19. Difference in dynamics of IgG responses with and without prior COVID‐19 may be due to the reactivation of Tmemory cells in response to cross reactive epitopes present due to natural infections.^44^ This fits with studies describing that hybrid immunity from vaccinations from a combination of prior infections and exposure, results in stronger immune responses.^45^

Our study has some limitations. The COVID‐19 vaccination rollout was rapid and on an emergency basis in our study population which comprised mainly, healthcare workers and older aged individuals populations. Due to the time taken for regulatory and ethical approvals for the study at the time of the urgent roll‐out of BBIBP‐CorV vaccination in Pakistan, we could not unfortunately determine baseline data exactly for the subjects in the BBIBP‐CorV vaccine study. However, this study population is the same where we earlier investigated antibody seroprevalence before vaccinations (conducted March–October 2020), where seropositivity to Spike was 35% and to RBD was 21.3% to RBD.^46^ As, many vaccinees here belonged to the larger study we expect that their baseline seroprevalence would be comparable. Neutralizing assays against SARS‐CoV‐2 were not conducted in this cohort. However, we have shown previously that sera samples positive for IgG to RBD had neutralizing activity against SARS‐CoV‐2.^47^ Another concern was that we could not collect regular samples from all subjects despite the study design due to lack of compliance of study subjects. This was due partly to increased hesitation on the part of study participants during the pandemic.

## 5 CONCLUSIONS

Overall, this study provides important insights into the immune activation in response to inactivated vaccines. In Pakistan, BBIBP‐CorV vaccination was shown to reduce hospitalization, mortality and symptomatic COVID‐19 in the older age group however, this was based on testing after 14 days of the second dose, or 6 weeks postvaccination.^48^ While BBIBP‐CorV vaccination is seen to reduce hospitalization and symptomatic COVID‐19, it is demonstrated that levels of IgG to RBD are lower than after other vaccinations and therefore, booster vaccinations are recommended.^49^ A recent case–control study from our center showed inactivated type of vaccines to be moderately effective in preventing symptomatic COVID‐19, but less effective than mRNA vaccines.^50^ We provide immunological insights into the effect of inactivated COVID‐19 vaccine type, BBIBP‐CorV. We observed an age‐dependent effect with reduced humoral and T cell responses in those aged 50 years and over, supporting a role for booster vaccinations in this group. Importantly the study illustrates the impact of prior COVID‐19 on boosting immune responses after vaccination. Our data also suggests, that regional variations may occur due to variable immunity from additional exposures. Hence, recommendations for COVID‐19 vaccinations should be made in the context of local immunity and ongoing transmission in the population.

## AUTHOR CONTRIBUTIONS

**Zahra Hasan**: Conceptualization; formal analysis; funding acquisition; investigation; methodology; project administration; supervision; validation; writing—original draft; writing—review and editing. **Kiran Iqbal Masood**: Formal analysis; investigation; methodology; project administration; validation; writing—original draft; writing—review and editing. **Shama Qaiser**: Data curation; formal analysis; methodology; validation; writing—original draft. **Erum Khan**: Methodology; resources; writing—original draft. **Areeba Hussain**: Data curation; investigation. **Zara Ghous**: Data curation; methodology. **Unab Khan**: Data curation; investigation; writing—original draft. **Maliha Yameen**: Data curation; formal analysis; methodology; validation. **Imran Hassan**: Data curation; investigation; resources. **Muhammad Imran Nasir**: Formal analysis; methodology. **Muhammad Farrukh Qazi**: Formal analysis; methodology. **Haris Ali Memon**: Data curation; methodology. **Shiza Ali**: Data curation; methodology. **Sadaf Baloch**: Data curation; methodology. **Zulfiqar A. Bhutta**: Investigation; resources. **Marc Veldhoen**: Methodology; writing—original draft. **J. Pedro Simas**: Methodology; resources. **Syed Faisal Mahmood**: Investigation; methodology; writing—original draft. **Kulsoom Ghias**: Funding acquisition; project administration; writing—original draft. **Rabia Hussain**: Formal analysis; methodology; validation; writing—review and editing.

## CONFLICT OF INTEREST STATEMENT

The authors declare no conflict of interest.

## ETHICS STATEMENT

This study was approved by the Ethical Review Committee of Aga Khan University (AKU) (project #2020‐5152‐11688).

## TRANSPARENCY STATEMENT

The lead author Zahra Hasan affirms that this manuscript is an honest, accurate, and transparent account of the study being reported; that no important aspects of the study have been omitted; and that any discrepancies from the study as planned (and, if relevant, registered) have been explained.

## Supporting information

Supporting information.

## Data Availability

All authors have read and approved the final version of the manuscript, Zahra Hasan had full access to all of the data in this study and takes complete responsibility for the integrity of the data and the accuracy of the data analysis. The authors confirm that the data supporting the findings of this study are available within the article and its supplementary materials.
