# Supplementary material for: Investigating the impact of prior COVID‐19 on IgG antibody and interferon γ responses after BBIBP‐CorV vaccination in a disease endemic population: A prospective observational study
Source: Health Sci Rep. 2023 Sep 8;6(9):e1521. doi: 10.1002/hsr2.1521 (PMC10486204; doi:10.1002/hsr2.1521)
Supplement: Supplementary file 1 — Supporting information. [file HSR2-6-e1521-s001.pdf]

Supplementary Figure 1. Overview of sampling after BBIBP-CorV adminstration

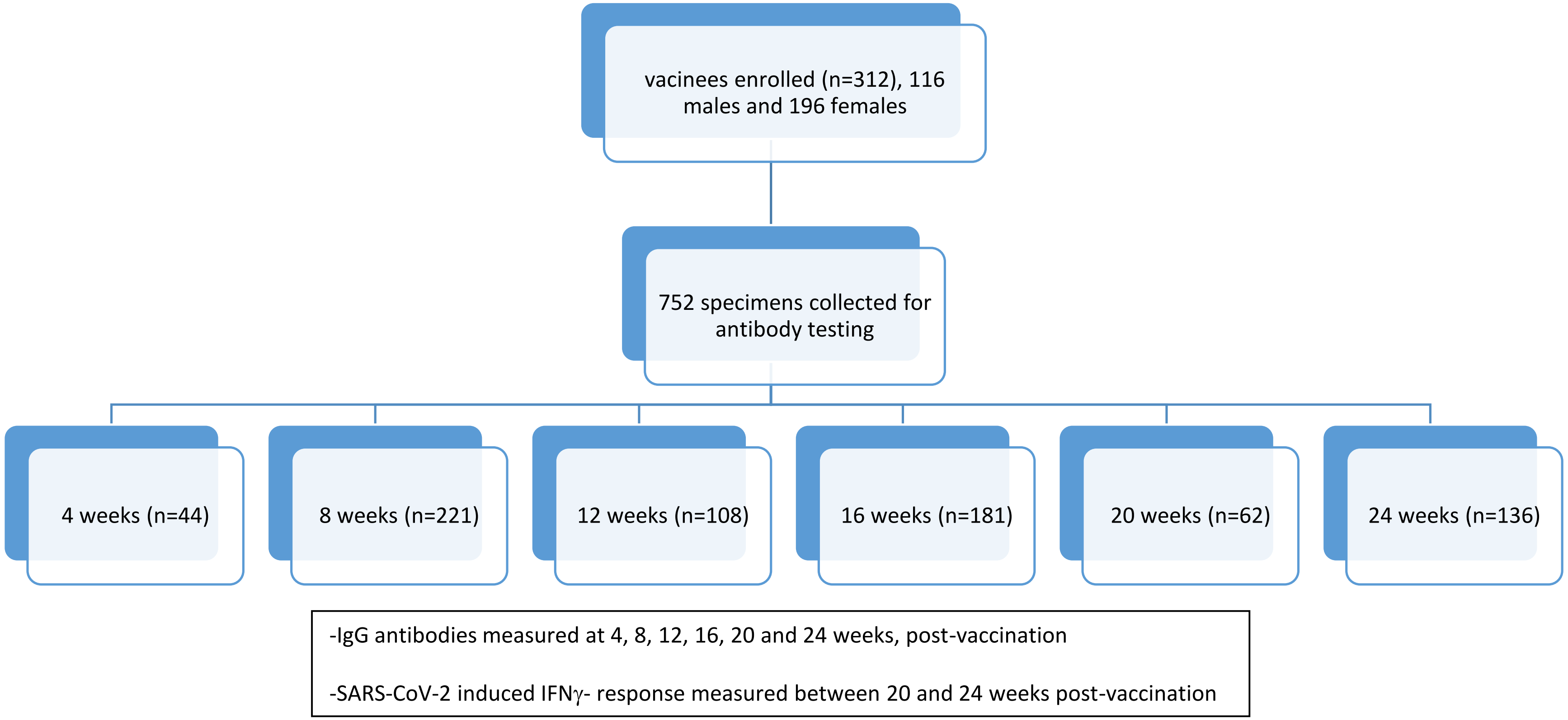

Supplementary Figure 2. Cellular response after BBIBP-CorV vaccination. Ninety-nine donors were sampled after vaccination. IFN-γ secreted by T cells was measured in plasma of whole blood cells stimulated with a commercial antigen assay containing peptides to SARS-CoV-2 spike Ag1, Ag2 and extended set (Ag3). IFN-γ was measured using the QuantIFERON, Qiagen, Cellestis, GMBH. Cut off for positive results was (0.15 IU/ml). A, IFN-γ secretion from each individual is depicted in response to antigen stimulation. Graphs show the geometric mean as a horizontal bar with 95%CI indicated by error bars. '*', denotes $p<0.05$. B, The percentage of individuals who had a positive IFN-γ response to antigen stimulation is shown.

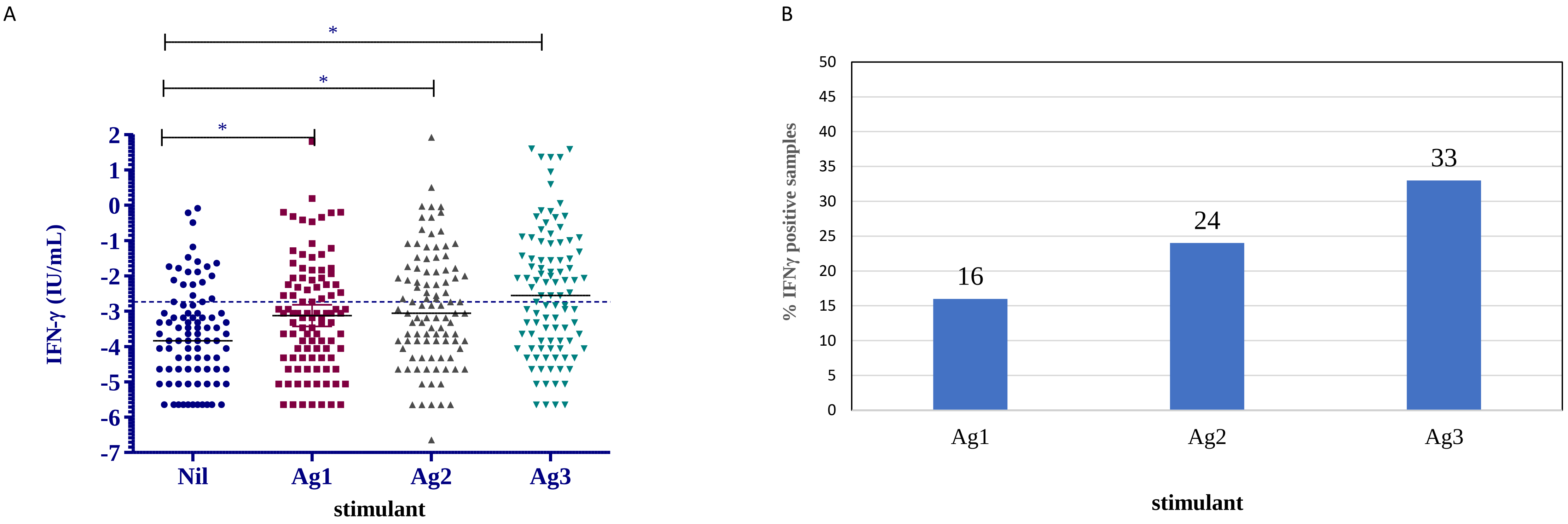

Supplementary Table 1. Descriptive results of SARS-CoV-2 antigen QuantiFERON assay

	nil	Ag1	Ag2	Ag3
Number of values	99	99	99	99
Minimum	0.0200	0.0200	0.0100	0.0200
25% Percentile	0.0400	0.0500	0.0700	0.0600
Median	0.0700	0.1200	0.1200	0.1700
75% Percentile	0.1200	0.2300	0.2800	0.4000
Maximum	0.9400	3.490	3.780	3.030
Mean	0.1195	0.2217	0.2574	0.3951
Std. Deviation	0.1559	0.4007	0.4396	0.6300
Std. Error	0.01566	0.04027	0.04418	0.06332
Lower 95% CI of mean	0.08841	0.1418	0.1697	0.2694
Upper 95% CI of mean	0.1506	0.3016	0.3451	0.5207
Sum	11.83	21.95	25.48	39.11

Supplementary Table 2. Multivariate analysis to determine relationship between IgG seropositivity to Spike protein in the context of age, gender and history of COVID19

	at 4 Weeks (N= 44)				at 8 Weeks (N= 221)				at 12 Weeks(N= 108)				at 16 Weeks (N= 180)				at 20 Weeks (N= 62)				at 24 Weeks (N= 136)			
	Positive	Negative	OR (CI)	P-value	Positive	Negative	OR (CI)	P-value	Positive	Negative	OR (CI)	P-value	Positive	Negative	OR (CI)	P-value	Positive	Negative	OR (CI)	P-value	Positive	Negative	OR (CI)	P-value
	N=27	N=17			N=201	N=20			N=83	N=25			N=139	N=41			N=53	N=9			N=126	N=10		
**History of COVID19**																								
Yes	12 (92%)	1 (8%)	12.8(1.48,110.79)	0.021	62 (95.4%)	3 (4.6%)	2.53(0.71,8.94)	0.15	29 (88%)	4 (12%)	2.82(0.88,9)	0.08	46 (85.2%)	8 (14.8%)	2.04(0.87,4.77)	0.1	16 (100%)	0 (0%)	-		43 (100%)	0 (0%)	-	
No	15 (48%)	16 (52%)	Ref		139 (89.1%)	17 (10.9%)	Ref		54 (72%)	21 (28%)	Ref		93 (73.8%)	33 (26.2%)	Ref		37 (80%)	9 (20%)	Ref		83 (89%)	10 (11%)	Ref	
**Age, years**																								
≤ 50	26 (65%)	14 (35%)	Ref		164 (97.0%)	5 (3.0%)	Ref		55 (90%)	6 (10%)	Ref		111 (81.6%)	25 (18.4%)	Ref		35 (88%)	5 (13%)	Ref		95 (96%)	4 (4%)	Ref	
>50	1 (25%)	3 (75%)	0.18(0.02,1.89)	0.153	37 (71.2%)	15 (28.8%)	0.08(0.03,0.22)	<0.001	28 (60%)	19 (40%)	0.16(0.06,0.45)	<0.001	28 (63.6%)	16 (36.4%)	0.39(0.19,0.84)	0.015	18 (82%)	4 (18%)	0.64(0.15,2.69)	0.545	31 (84%)	6 (16%)	0.22(0.06,0.82)	0.024
**Gender**																								
Male	8 (53%)	7 (47%)	Ref		69 (84.1%)	13 (15.9%)	Ref		26 (62%)	16 (38%)	Ref		44 (72.1%)	17 (27.9%)	Ref		25 (81%)	6 (19%)	Ref		47 (87%)	7 (13%)	Ref	
Female	19 (66%)	10 (34%)	1.66(0.47,5.93)	0.433	132 (95.0%)	7 (5.0%)	3.55(1.36,9.31)	0.01	57 (86%)	9 (14%)	3.9(1.52,9.97)	0.005	95 (79.8%)	24 (20.2%)	1.53(0.75,3.13)	0.245	28 (90%)	3 (10%)	2.24(0.51,9.91)	0.288	79 (96%)	3 (4%)	3.92(0.97,15.9)	0.056

Supplementary Table 3. Multivariate analysis to determine relationship between IgG seropositivity to RBD protein in the context of age, gender and history of COVID19

	at 4 Weeks (N= 44)				at 8 Weeks (N= 221)				at 12 Weeks(N= 108)				at 16 Weeks (N= 180)				at 20 Weeks (N= 62)				at 24 Weeks (N= 136)			
	Positive	Negative	OR (CI)	P-value	Positive	Negative	OR (CI)	P-value	Positive	Negative	OR (CI)	P-value	Positive	Negative	OR (CI)	P-value	Positive	Negative	OR (CI)	P-value	Positive	Negative	OR (CI)	P-value
	N=22	N=22			N=150	N=71			N=76	N=32			N=137	N=43			N=50	N=12			N=119	N=17		
**History of COVID19**																								
Yes	11 (85%)	2 (15%)	10(1.87,53.48)	0.007	48 (73.8%)	17 (26.2%)	1.49(0.79,2.85)	0.221	28 (85%)	5 (15%)	3.15(1.09,9.11)	0.034	46 (85.2%)	8 (14.8%)	2.21(0.95,5.15)	0.066	16 (100%)	0 (0%)	-		43 (100%)	0 (0%)	-	
No	11 (35%)	20 (65%)	Ref		102 (65.4%)	54 (34.6%)	Ref		48 (64%)	27 (36%)	Ref		91 (72.2%)	35 (27.8%)	Ref		34 (74%)	12 (26%)	Ref		76 (82%)	17 (18%)	Ref	
**Age, years**																								
≤ 50	21 (53%)	19 (48%)	Ref		120 (71.0%)	49 (29.0%)	Ref		50 (82%)	11 (18%)	Ref		107 (78.7%)	29 (21.3%)	Ref		34 (85%)	6 (15%)	Ref		91 (92%)	8 (8%)	Ref	
>50	1 (25%)	3 (75%)	0.3(0.03,3.15)	0.317	30 (57.7%)	22 (42.3%)	0.56(0.29,1.06)	0.074	26 (55%)	21 (45%)	0.27(0.11,0.65)	0.003	30 (68.2%)	14 (31.8%)	0.58(0.27,1.24)	0.159	16 (73%)	6 (27%)	0.47(0.13,1.69)	0.248	28 (76%)	9 (24%)	0.27(0.1,0.78)	0.015
**Gender**																								
Male	6 (40%)	9 (60%)	Ref		54 (65.9%)	28 (34.1%)	Ref		24 (57%)	18 (43%)	Ref		43 (70.5%)	18 (29.5%)	Ref		24 (77%)	7 (23%)	Ref		44 (81%)	10 (19%)	Ref	
Female	16 (55%)	13 (45%)	1.85(0.52,6.55)	0.343	96 (69.1%)	43 (30.9%)	1.16(0.65,2.07)	0.622	52 (79%)	14 (21%)	2.79(1.19,6.51)	0.018	94 (79.0%)	25 (21.0%)	1.57(0.78,3.19)	0.207	26 (84%)	5 (16%)	1.52(0.42,5.43)	0.522	75 (91%)	7 (9%)	2.44(0.86,6.86)	0.092
